# How to remove the influence of trace water from the absorption spectra of SWNTs dispersed in ionic liquids

**DOI:** 10.3762/bjnano.2.69

**Published:** 2011-09-30

**Authors:** Juan Yang, Daqi Zhang, Yan Li

**Affiliations:** 1 Beijing National Laboratory for Molecular Sciences, State Key Laboratory of Rare Earth Materials Chemistry and Applications, Key Laboratory for the Physics and Chemistry of Nanodevices, College of Chemistry and Molecular Engineering, Peking University, Beijing 100871, China

**Keywords:** absorption spectra, ionic liquids, quantitative analysis, single-walled carbon nanotubes

## Abstract

Single-walled carbon nanotubes (SWNTs) can be efficiently dispersed in the imidazolium-based ionic liquids (ILs), at relatively high concentration, with their intrinsic structure and properties retained. Due to the hygroscopicity of the ILs, water bands may be introduced in the absorption spectra of IL-dispersed SWNTs and cause problems in spectral deconvolution and further analysis. In order to remove this influence, a quantitative characterization of the trace water in [BMIM]^+^[PF_6_]^−^ and [BMIM]^+^[BF_4_]^−^ was carried out by means of UV–vis-NIR absorption spectroscopy. A simple yet effective method involving spectral subtraction of the water bands was utilized, and almost no difference was found between the spectra of the dry IL-dispersed SWNT samples treated under vacuum for 10 hours and the spectra of the untreated samples with subtraction of the pure water spectrum. This result makes it more convenient to characterize SWNTs with absorption spectra in the IL-dispersion system, even in the presence of trace amount of water.

## Introduction

The so-called room-temperature ionic liquids (ILs) are a group of room-temperature molten salts that are composed of specific cations and anions [1–2]. Compared to conventional volatile organic solvents, they are nonpolluting, recyclable green solvents with remarkable physical and chemical properties, including low melting points, nondetectable vapor pressure, excellent stability, etc. [1–3]. In addition, by varying the structures of the component cations or anions the properties of ILs can be easily adjusted. Due to all the above advantages, ILs have attracted significant attention in many research areas such as electrochemistry [4–5], and chemical reactions and separations [6–9].

In 2003, Fukushima et al. [10] found that by mixing together and mechanically grinding the single-walled carbon nanotubes with imidazolium-based ILs, a thermally stable bucky gel can be formed with SWNTs untangled from the heavily entangled bundles. Since the poor solubility and low dispersibility of SWNTs in conventional solvents have hindered the processing and applications of SWNTs for more than a decade, this phenomenal discovery showed a new direction for the dispersal of SWNTs with high concentrations (~1 wt %), which are about two or three orders of magnitude higher than other suspension methods, including surfactant dispersion [11–12], DNA wrapping [13–14], polymer wrapping [15], and sidewall covalent functionalization [16–17]. As it does not involve any rigorous sonication, centrifugation, or chemical reaction, the structure of the SWNTs is not damaged. Moreover, a previous study [18] has shown that the electronic structure and properties of SWNTs are retained since there is no strong interaction, but only weak van der Waals interaction, between SWNTs and ILs. Therefore, imidazolium-based ILs are ideal media for the investigation of the properties and applications of SWNTs.

As it is well known that ILs are hygroscopic and that the amount of water absorbed in ILs can significantly affect the physical properties, such as polarity, viscosity, conductivity, and solubility [19–21], much research [21–22] have been carried out to study the states of water dissolved in ILs at the molecular level in order to achieve better understanding of ILs. It was found that the up-taken water interacts strongly through hydrogen bonding with the anions of the ILs, and for 1-butyl-3-methylimidazolium ([BMIM]^+^)-based ILs, [BF_4_]^−^ provides stronger interactions than [PF_6_]^−^ and more water is absorbed in [BMIM]^+^[BF_4_]^−^ than in [BMIM]^+^[PF_6_]^−^ [22].

In the case of IL-dispersed SWNTs, the presence of water not only changes the properties of the IL but also affects the spectroscopic characterization of the dispersed SWNTs. The optical transitions of SWNTs occur when the energy of the incident radiation matches the energy gap between the van Hove singularities of SWNTs. For HiPco samples, in which the diameter of the nanotubes is about 0.7–1.1 nm, the E_11_ and E_22_ transitions of the semiconducting nanotubes are in the ranges of 850–1600 nm and 600–800 nm, respectively, while the E_11_ transitions of the metallic nanotubes are of 400–650 nm [23]. As the E_11_ transitions of semiconducting nanotubes are of the lowest energy and do not overlap with higher energy transitions, the deconvolution of the absorption bands in this region with respect to their chiralities is of great importance in the quantitative analysis of bulk SWNT samples. However, because water has strong absorption bands in the near-infrared (NIR) region where the E_11_ of semiconducting SWNTs lies, even a trace amount of water dissolved in an IL may introduce notable peaks in the SWNTs absorption spectra, which will affect the deconvolution and quantitative analysis significantly. Therefore, treatment of the ILs under high vacuum, immediately before taking the spectra, is necessary to reduce the peaks introduced by the trace amount of water. Even so, the water bands are still inevitable because ILs will absorb water from the atmosphere during the period of time it takes to collect the spectra. In order to correct for the influence of water in the absorption spectra of SWNTs dispersed in ILs and furthermore to avoid an inconvenient sample treatment procedure, a simple but effective method is needed.

In this paper, a study of the UV-vis-NIR spectra of [BMIM]^+^ based ILs with different amounts of added water is described, and a quantitative characterization of the spectra with respect to the water concentrations is made. A spectral-subtraction method is used to remove the water bands from the absorption spectra of IL-dispersed SWNTs and the results are compared with the corresponding spectra of water-free samples. The exact amount of water taken up in the untreated sample is calculated consequently.

## Experimental

The [BMIM]^+^[PF_6_]^−^ and [BMIM]^+^[BF_4_]^−^ ILs were purchased from Henan Lihua Pharmaceutical Co. Ltd., China. The as-received ILs were first treated under 10^−5^ Pa vacuum for 10 h to remove the absorbed water. Different amount of water (produced by Millipore SimPak 1, resistivity ≥18.2 MΩ cm) was then added to the dry ILs through a microsyringe. The water concentrations were calculated by the volume of added water and the weights of the dry ILs.

The SWNT suspensions in ILs were prepared by mechanically grinding ~0.1 mg HiPco SWNTs and ~8.0 g untreated ILs together in an agate mortar for 20 min. The as-prepared samples (“untreated” samples) were used directly for spectral measurements. As comparison, the mixtures were then treated under 10^−5^ Pa vacuum for 10 h and the corresponding spectra of the “dry” samples were collected with dehydrates present in the sample chamber to maximally avoid the absorption of water vapor from the atmosphere.

The UV–vis-NIR absorption spectra were collected in a cell with a 1.0 cm path length with a PerkinElmer Lambda 950 spectrophotometer. A scan rate of 140 nm/min with a step size of 0.5 nm was typically used. The spectra for ILs were recorded in the 300–2000 nm region while those for SWNTs-ILs were measured in the 300–1800 nm region. The program Origin 8.0 was used for data analysis.

## Results and Discussion

**Figure 1:**
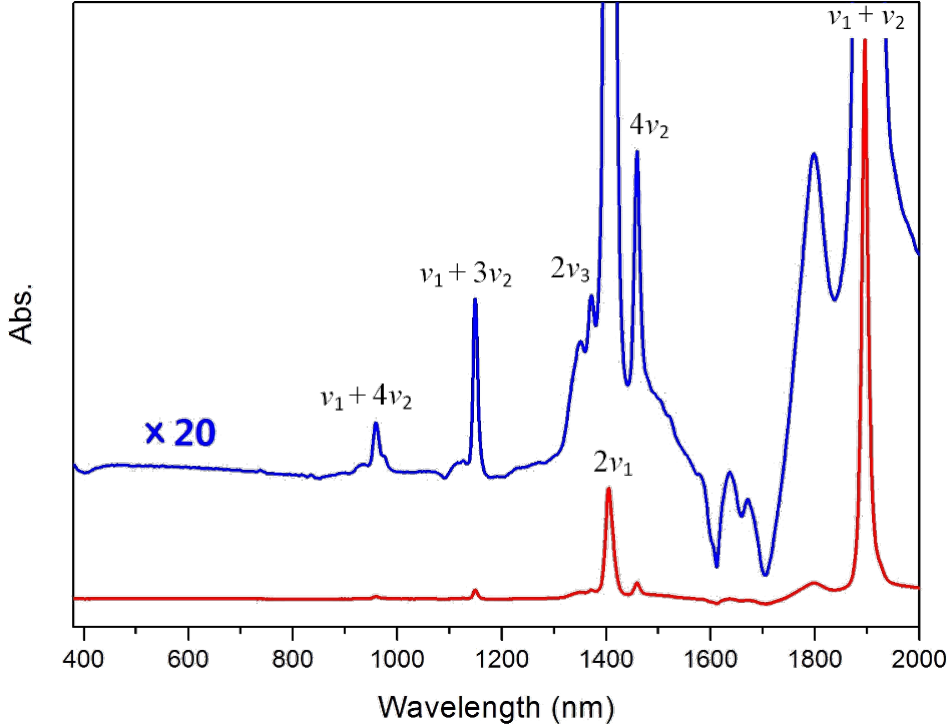
The UV–vis-NIR spectra of water in [BMIM]^+^[PF_6_]^−^ with dry [BMIM]^+^[PF_6_]^−^ used as a reference. The blue spectrum is a 20-times magnification of the red original spectrum in order to enlarge the weak signals.

Figure 1 illustrates the UV–vis-NIR spectra of [BMIM]^+^[PF_6_]^−^ with a water concentration of 0.266 M, where dry [BMIM]^+^[PF_6_]^−^ is used as a reference and thus its absorption is subtracted out automatically. The blue spectrum is a 20-times magnification of the red original spectrum in order to enlarge the weak signals. Since HiPco SWNTs only show absorption bands up to 1800 nm, it is the 300–2000 nm spectral region that we are most interested in. The negative bands below 450 nm and in the spectral region of 1600–1750 nm are due to the absorption of [BMIM]^+^ [PF_6_]^−^, which was previously reported [22].

In the water spectra two main features are observed at 1405 and 1896 nm, and can be attributed to the first overtone of the O–H symmetric stretching vibration (2*ν*_1_) and the combination of the O–H symmetric stretching and the angle bending vibrations (*ν*_1_ + *ν*_2_), respectively. The weak band at 1372 nm is assigned as the first overtone of the O–H antisymmetric stretching vibration (2*ν*_3_). Thus the three fundamental vibrations of water in [BMIM]^+^[PF_6_]^−^ can be calculated as *ν*_1_ = 3559 cm^−1^, *ν*_2_ = 1715 cm^−1^, and *ν*_3_ = 3644 cm^−1^, respectively. These numbers match very nicely with other bands in the spectra at 1460, 1149, and 959 nm, with the assignments as 4*ν*_2_, *ν*_1_ + 3*ν*_2_, and *ν*_1_ + 4*ν*_2_, respectively. The *ν*_1_ + 2*ν*_2_ band is expected to be at 1431 nm but is not observed in the spectra. It may be that it is covered by the much more intense band at 1405 nm.

It is well known that for free water molecules the three fundamental vibrational frequencies are *ν*_1_ = 3652 cm^−1^, *ν*_2_ = 1595 cm^−1^, and *ν*_3_ = 3756 cm^−1^ [24]. In [BMIM]^+^[PF_6_]^−^, water interacts strongly with the [PF_6_]^−^ anion through hydrogen bonding and the structure of water is changed consequently, which leads to the vibrational frequency shifts. In particular, due to the hydrogen bonding between the H atom in water and the F atom in [PF_6_]^−^, the O–H bonds are weakened, and thus the two stretching vibrations move to lower wavenumbers, as expected. The angle bending vibration, on the other hand, shifts to higher frequency because of the increased force constant arising from a more rigid H–O–H structure in the presence of hydrogen bonding.

**Figure 2:**
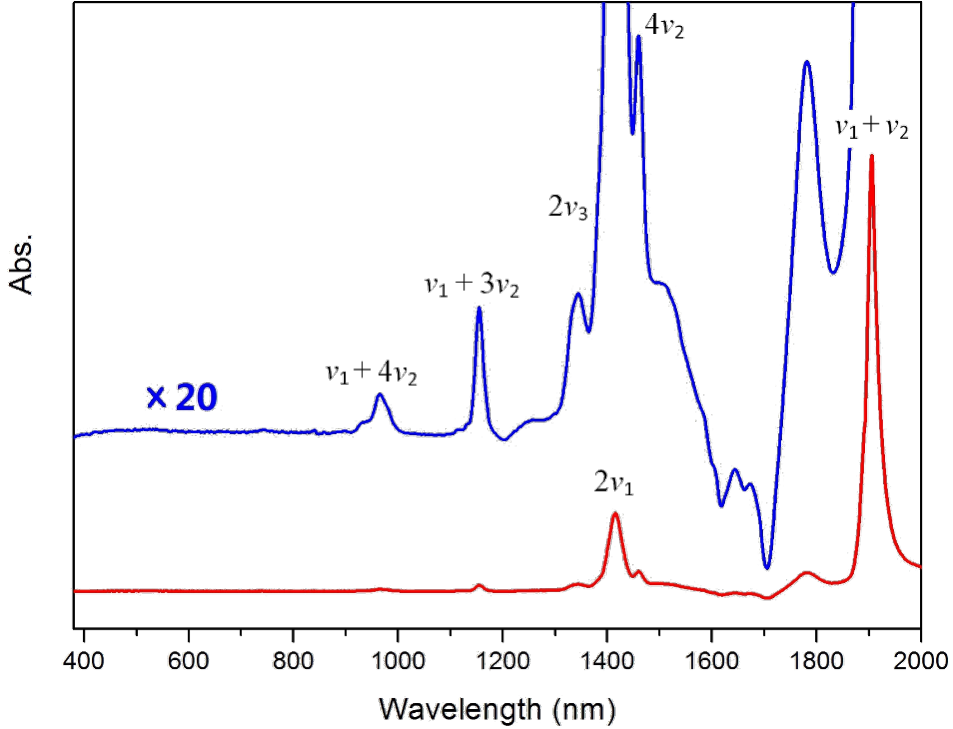
The UV–vis-NIR spectra of water in [BMIM]^+^[BF_4_]^−^ with dry [BMIM]^+^[BF_4_]^−^ used as reference. The blue spectrum is a 20-times magnification of the red original spectrum in order to enlarge the weak signals.

The strength of the interactions between ILs and water depends mainly on the anions, therefore, ILs with different types of anions have different influence on the structural changes of the water molecule. The absorption spectra of water in [BMIM]^+^[BF_4_]^−^ is shown in Figure 2, in which the blue spectrum is again the 20-times magnification of the red original spectrum. From the 2*ν*_1_, 2*ν*_3_, and *ν*_1_ + *ν*_2_ bands observed at 1416, 1383, and 1906 nm the corresponding fundamental vibrational frequencies of water can be calculated as *ν*_1_ = 3531 cm^−1^, *ν*_2_ = 1716 cm^−1^, and *ν*_3_ = 3615 cm^−1^, respectively. The lower O–H stretching and slightly higher H–O–H bending vibrational frequencies suggest stronger interactions of water with [BMIM]^+^[BF_4_]^−^ than with [BMIM]^+^[PF_6_]^−^.

**Figure 3:**
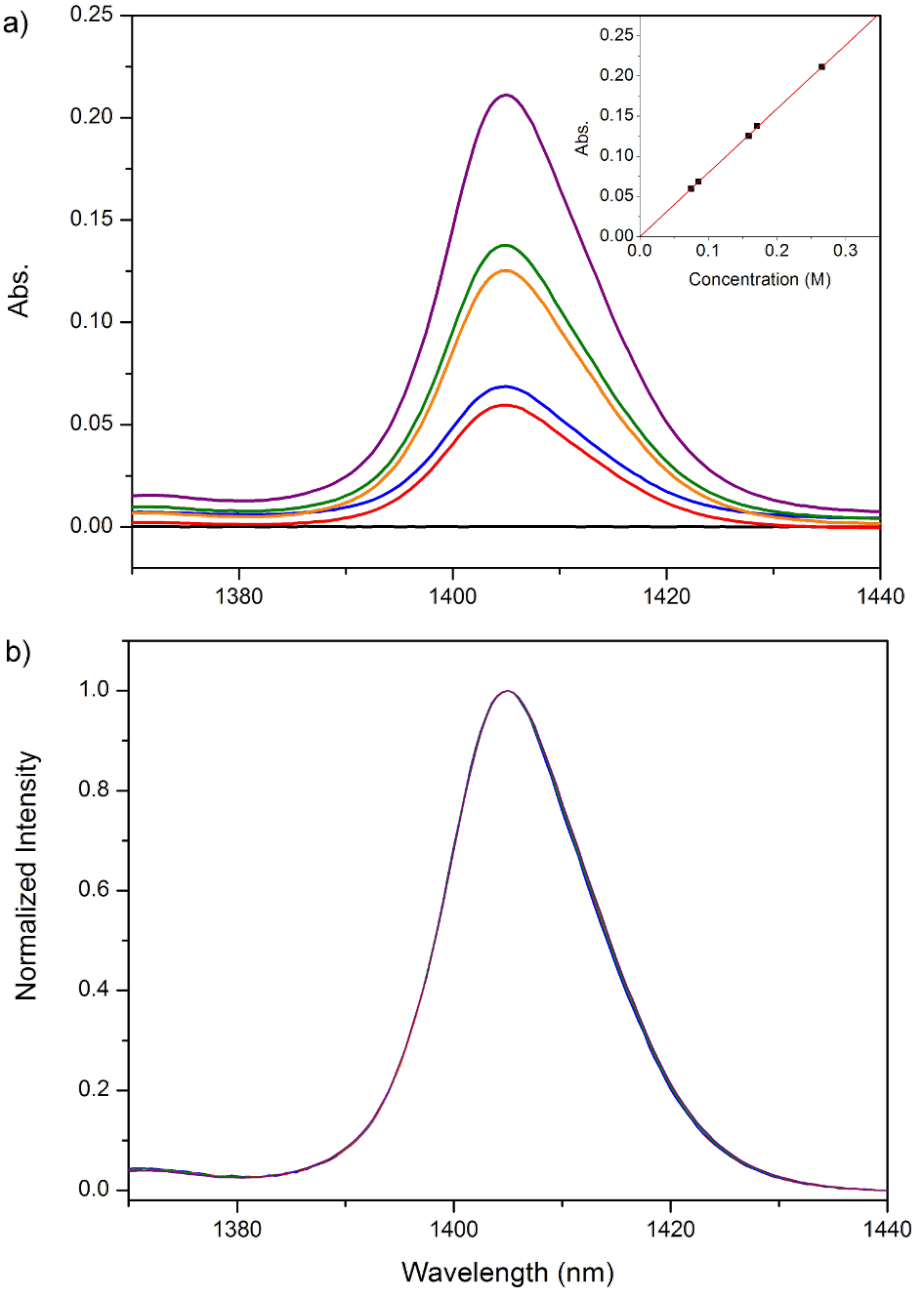
a) The absorption bands at 1405 nm with different amounts of water (from bottom to top: 0.000, 0.075, 0.086, 0.159, 0.171, and 0.266 M) added to dry [BMIM]^+^[PF_6_]^−^. The inset plots the linear fitting of band intensity vs. water concentration. b) Intensity normalization of spectra in a).

In Figure 3a the absorption bands at 1405 nm with different amounts of water (0.000, 0.075, 0.086, 0.159, 0.171, and 0.266 M) added to the dry [BMIM]^+^[PF_6_]^−^ are plotted. It is clear that with the increased water concentration the band intensity increases concomitantly. After intensity normalization, all five bands exhibit identical frequency and shape, as shown in Figure 3b, indicating that the molecular state of water does not change in the experimental concentration range. The diagram of band intensity versus water concentration is plotted in the inset of Figure 3a and is fitted by a linear relationship. The intercept is fixed to be zero during the iteration and the resulting linear function is given by

[1]
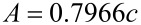


where *A* is the absorbance at 1405 nm and *c* is the water molar concentration (in M) in [BMIM]^+^[PF_6_]^−^. An *R*^2^ of 0.99993 indicates excellent linear relationship between *A* and *c*. Although the peak areas are suppose to be used in the spectral fitting, the peak intensities could be used instead in this case because both the band shape and position remain unchanged.

**Figure 4:**
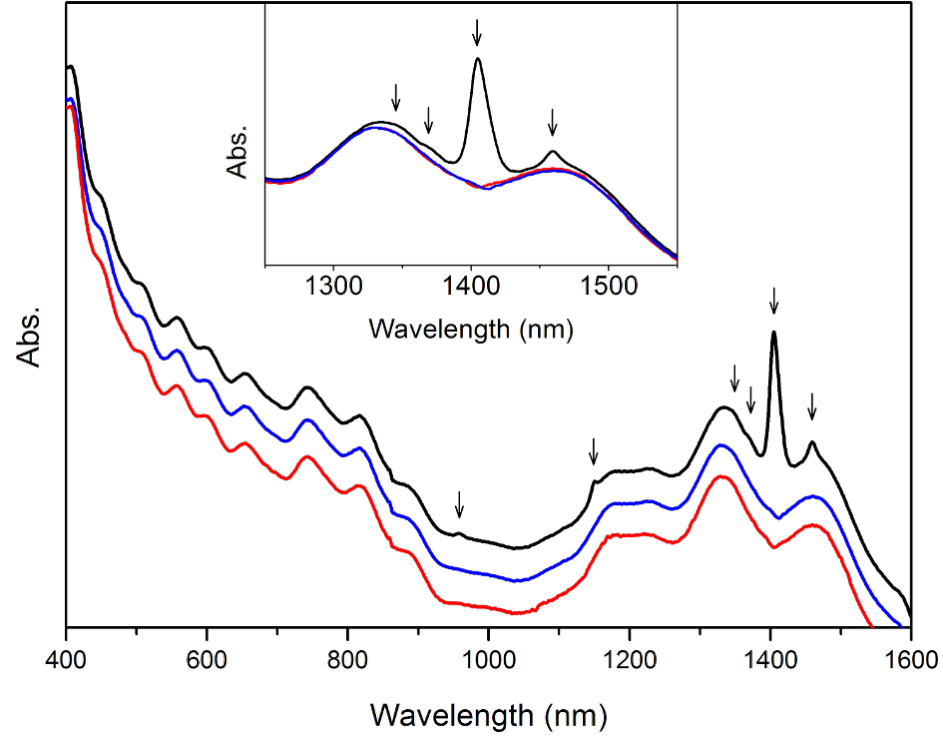
Absorption spectra of [BMIM]^+^[PF_6_]^−^-dispersed SWNTs without treatment (black curve), after subtraction of water spectrum (blue curve), and treated under vacuum for 10 hours (red curve). The spectra are offset arbitrarily in the vertical direction for clarity. The inset shows the partially enlarged spectra. Arrows indicate the positions of water bands.

The absorption spectrum of the untreated [BMIM]^+^[PF_6_]^−^-dispersed SWNTs is shown as the black curve in Figure 4, with arrows indicating where the water peaks lie. The well-resolved electronic absorption bands of SWNTs demonstrate good dispersion of nanotubes in this IL. The intensity of the water band at 1405 nm is even higher than that of the nanotube absorption bands, so the deconvolution of the two broad bands close to 1400 nm with respect to semiconducting nanotube chiralities will be affected significantly by this intense water band. In order to remove this effect as well as to quantify the amount of water taken up by the untreated sample, a spectral subtraction method was utilized. To obtain the best fit with the spectrum of the water-free sample, given by the red curve in Figure 4, a specific multiplying parameter was used to scale the water spectrum in Figure 1, which was then subtracted from the spectrum of the untreated sample, the result of which is given by the blue curve. It is clearly shown in Figure 4 that not only the intense band at 1405 nm but also other weak bands can be subtracted out from the spectra at the same time with this best-fitting parameter. The excellent agreement of the blue and red curves verifies the feasibility of this spectral-subtraction method. The water concentration in the untreated sample can be calculated from Equation 1 as 0.0304 M and the corresponding amount of water taken up is 6.40 × 10^3^ ppm.

**Figure 5:**
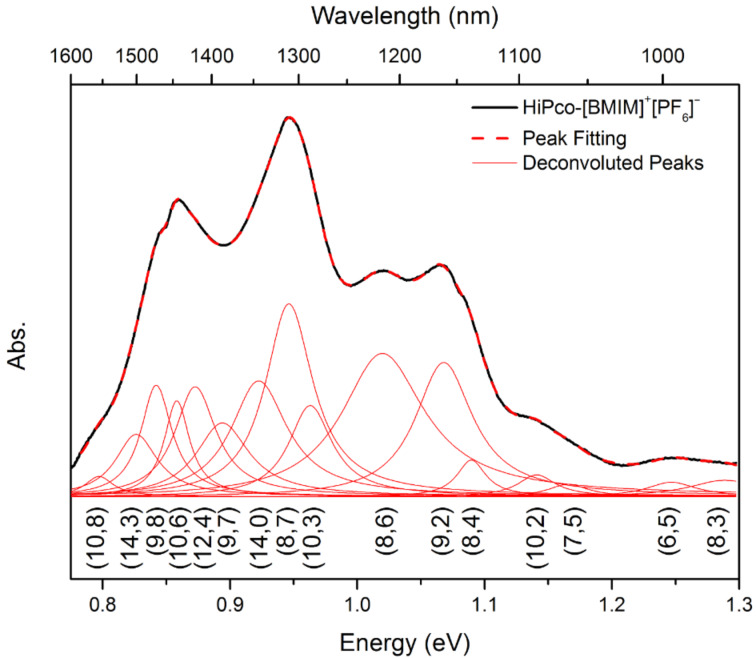
Deconvolution of the baseline-corrected absorption spectra of [BMIM]^+^[PF_6_]^−^-dispersed SWNTs in the semiconducting nanotube E_11_ region with the solid black curve denoting the baseline-corrected spectrum, the dashed red curve denoting the fitting spectrum, and the solid red curves denoting the deconvoluted individual peaks corresponding to different chiralities of the semiconducting nanotubes.

After removal of the water bands from the absorption spectra of SWNTs dispersed in [BMIM]^+^[PF_6_]^−^, further analysis of the spectra can be carried out to obtain quantitative information about the bulk SWNT samples. As an example, Figure 5 illustrates the deconvolution of the baseline-corrected spectra in the E_11_ transition region of the semiconducting nanotubes, with the solid black curve as the baseline-corrected spectrum, the dashed red curve as the fitting spectrum, and the solid red curves as the deconvoluted individual peaks corresponding to different chiralities of the semiconducting nanotubes, as indicated in the figure. Compared to surfactant SDS-dispersed HiPco SWNTs [23], an average of 30 meV red-shift in energy is observed in the semiconducting nanotube E_11_ region and this can be attributed to changes in the surrounding dielectric environment caused by [BMIM]^+^[PF_6_]^−^.

**Figure 6:**
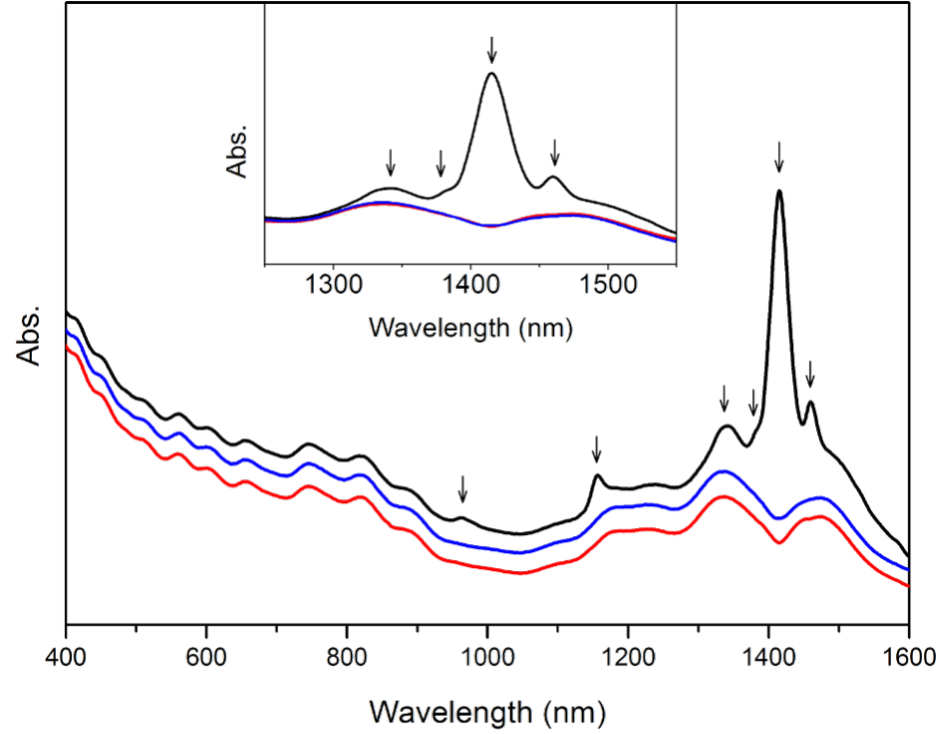
Absorption spectra of [BMIM]^+^[BF_4_]^−^-dispersed SWNTs without treatment (black curve), after subtraction of water spectrum (blue curve), and treated under vacuum for 10 hours (red curve). The spectra are offset arbitrarily in the vertical direction for clarity. The inset shows the partially enlarged spectra. Arrows indicate the positions of water bands.

A similar spectral-subtraction method was carried out with SWNTs dispersed in [BMIM]^+^[BF_4_]^−^ and the resulting spectra are plotted in Figure 6. The agreement of the spectrum after subtraction of water with that of the water-free sample is also excellent, indicating the validity of this method for different types of ILs.

## Conclusion

In this paper we have demonstrated a simple yet effective method for the spectral subtraction of the influence of trace water taken up by the ILs [BMIM]^+^[PF_6_]^−^ and [BMIM]^+^[BF_4_]^−^ on the absorption spectra of IL-dispersed SWNTs. The resulting spectra are in very good agreement with the spectra of water-free samples treated under high vacuum for 10 h. By utilizing this spectral-subtraction method, the additional step of sample treatment under vacuum can be avoid. The spectra after subtraction can be used directly for deconvolution and further quantitative analysis. This makes the characterization of SWNTs by analysis of absorption spectra more convenient in the IL-dispersion system.
